# Treatment Patterns for and Characteristics of Headache in Children and Adolescents Aged 6–17 Years in Japan: A Retrospective Cross-Sectional and Longitudinal Analysis of Health Insurance Claims Data

**DOI:** 10.3390/life14010096

**Published:** 2024-01-08

**Authors:** Masahito Katsuki, Yasuhiko Matsumori, Taisuke Ichihara, Yuya Yamada, Shin Kawamura, Kenta Kashiwagi, Akihito Koh, Tetsuya Goto, Kazuma Kaneko, Naomichi Wada, Fuminori Yamagishi

**Affiliations:** 1 Department of Neurosurgery, Suwa Red Cross Hospital, Suwa 392-0027, Japan; 2 Headache Outpatient, Suwa Red Cross Hospital, Suwa 392-0027, Japan; 3 Sendai Headache and Neurology Clinic, Sendai 982-0014, Japan; 4 Japan System Techniques Co., Ltd. (JAST), Minato-ku 108-8288, Japan; 5 Department of Neurosurgery, Itoigawa General Hospital, Itoigawa 941-0006, Japan; 6 Department of Neurology, Itoigawa General Hospital, Itoigawa 941-0006, Japan; 7 Department of Neurology, Suwa Red Cross Hospital, Suwa 392-0027, Japan; 8 Department of Surgery, Itoigawa General Hospital, Itoigawa 941-0006, Japan

**Keywords:** migraine, pediatric, acute mediations, triptans, medication overuse headache

## Abstract

Objective: To investigate the prescription patterns for patients aged 6–17 years with headaches in the REZULT database. Methods: We cross-sectionally investigated (Study 1) the pattern of prescription and the proportion of triptan overprescription (≥30 tablets/90 d of triptans) among patients diagnosed with headaches in 2020. Next, we longitudinally studied patients (Study 2) for more than two years from the initial headache diagnosis (July 2010 to April 2022). The number of prescribed tablets was counted every 90 days. Results: In Study 1, headache diagnoses were assigned to 62,568 of 543,628 (11.51%) patients, and 1524 of 62,568 (2.44%) patients received acute medication. Single nonsteroidal anti-inflammatory drugs and triptans were prescribed to 620/624 (99.36%) and 5/624 (0.80%) of patients aged 6–11 years, respectively, and 827/900 (91.89%) and 91/900 (10.11%) of patients aged 12–17 years, respectively. Triptan overprescription was observed in 11/96 (11.46%) patients, and 5/11 (45.45%) of those patients received prophylactic medication. In Study 2, 80,756/845,470 (9.55%) patients aged 6–17 years were diagnosed with headaches that persisted for at least two years. Over two years, 44/80,756 (0.05%) patients were overprescribed triptans, and 3408/80,756 (4.22%) patients were prescribed prophylaxis on at least one occasion. Conclusions: Based on real-world data, the appropriate use of prophylactic treatment is still problematic. Overprescription of triptans was observed, although the number of patients was small.

## 1. Introduction

Primary headaches are a widespread public health problem. The prevalence of migraine is approximately 12%, and tension-type headache (TTH) occurs in up to 50% of the population [1,2]. Inappropriate acute medication use can cause medication overuse headache (MOH), with a prevalence of approximately 1–2% [3]. Headache disorders significantly impede the daily functioning of patients [4,5,6,7,8,9,10,11,12,13] and are a burden for children and adolescents. A study conducted in a Japanese city with 40,000 residents revealed that the prevalence rates of headaches, migraines, and MOHs in children and adolescents were 36.44%, 9.48%, and 0.44%, respectively. Among the 907 children and adolescents with headaches in the study, 638 (70.34%) had experienced a depressed mood due to headaches, 630 (69.46%) endured headaches, 386 (42.56%) left school early, 353 (38.92%) were absent from school, 337 (37.16%) had difficulty attending class, and 291 (32.08%) had difficulty in after school activities due to headaches. However, only 314 (34.62%) patients consulted a physician [14]. Similar trends have been reported worldwide [12,15].

Managing primary headaches involves the use of acute medications to treat headache attacks and prophylactic medications to decrease the occurrence and intensity [16]. Inappropriate prescription without prophylactic medication for severe headache disorders can increase the likelihood of developing resistance to treatment, experiencing additional health conditions, developing chronic migraines, and encountering medication overuse headaches (MOHs) [17,18]. However, there is a shortage of headache specialists, which leads to the management of primary headaches by general practitioners, family physicians, and pediatricians on a global scale [19,20,21,22]. As a result, the worrying issue of excessive prescription of acute medications without the concurrent use of preventive medications arises. In Japan, only 19 pediatricians specialize in headache management, and this is accompanied by a lack of suitable prescriptions in pediatrics.

Recently, data-based research has been published on National Health Insurance claims using numerous data sets from adults with headaches [23,24,25,26,27,28]. However, few of these studies have been conducted in Japan [29,30,31]. Furthermore, only two studies have used data from health insurance claims on the prevalence of headaches in children and adolescents [32,33]; this has not yet been carried out in Japan. Big data research can shed light on physician prescription patterns in Japan. Therefore, this study investigated a health insurance database to clarify the treatment patterns for headaches among children and adolescents aged 6–17 years.

## 2. Materials and Methods

Every individual residing in Japan must be provided health insurance by employers and local community insurers. In addition, the government has established cost schedules for healthcare services [29]. Receipts from health insurance providers that catered to employees were documented in the REZULT database, encompassing data from over 8 million patients. This information was collected by Japan System Techniques Co., Ltd. in Tokyo, Japan (https://www.jastlab.jast.jp/rezult_data/, accessed on 1 October 2023). The REZULT database included patients aged 6–11 years, corresponding to 5.51% of the Japanese population in that age bracket, whereas 12–17-year-olds constituted 4.89% of the sample. This database contains information such as consultation and treatment dates, the name of the disease, patient age, biological sex, and details regarding medical treatment, including prescriptions and information about the medical facility. Each patient enrolled in the database was assigned a unique and confidential identification number, allowing comprehensive monitoring of prescription patterns over time. 

In Japan, both combined pain relievers and single pain-relief medications are available for purchase without prescription (over-the-counter (OTC) medications). However, the sale of triptans and preventive migraine medications, such as antihypertensive drugs, antiepileptic drugs, and antidepressants as OTC medications is not allowed. Prophylactic treatment and triptans can only be prescribed for patients clinically diagnosed with migraine. Single and combined nonsteroidal anti-inflammatory drugs (NSAIDs) can be prescribed for headaches that do not necessarily lead to a migraine diagnosis. NSAIDs are also prescribed for other persistent pain conditions, such as lower back pain. However, tracking the conditions for which these drugs were prescribed is impossible. 

No data were available on the departments visited by patients with headaches. Pediatrics is the most commonly visited department for children under 15 years of age. General practice, neurology, and neurosurgery are the most frequently visited departments for this group. 

### 2.1. Study 1 (Cross-Sectional Study): Prescription Pattern for Headache Patients

Using the REZULT database, we extracted the data for patients aged 6–17 years with a 1-year continued diagnosis of “headache (International Statistical Classification of Diseases and Related Health Problems (ICD)-10 code R51)” or “Other headache syndromes” (ICD-10 code G44) in 2020. We included those who had received acute medications to examine trends in the usage of acute and prophylactic medications. The presence or absence of a diagnosis of “migraine” (identified by the ICD-10 code G43) was not considered. We did not use the International Classification of Headache Disorders, 3rd edition criteria (ICHD-3) because insurance claims are rarely based on ICHD-3 in Japan. 

Acute treatment was defined as the prescription of a single nonsteroidal anti-inflammatory drug (NSAID), a combination of NSAIDs, or triptans. Prophylactic treatments included the prescription of specific medications, including lomerizine, propranolol, valproic acid, amitriptyline, verapamil, and Japanese herbal Kampo medicine, as described in the Japanese guidelines [16]. Supplementary File S1 contains the codes for the drug price list. In Japan, topiramate cannot be used for migraine prophylaxis. The number of acute medication tablets prescribed between January 2020 and December 2020 was recorded and the percentage of cases involving excessive prescriptions was assessed.

Overprescription was defined according to ICHD-3 criteria as follows: the use of triptans or a combination of NSAIDs on more than 10 days (tablets) per month for three months (≥30 tablets/90 days (tbl/90 d)); the use of a single NSAID on more than 15 days (tablets) per month (≥45 tbl/90 d); or any combination of single or combined NSAIDs or triptans totaling ≥30 tbl/90 d. However, single NSAIDs were occasionally provided in forms like syrup or fine granules for children instead of tablets. Due to this variation, the overprescription of a single NSAID and overprescription of multiple drugs could not be calculated and were not the primary focus of this study. Furthermore, since determining the exact number of days for which acute medications were used during the study period was impossible, the count of “number of tablets per 90 days” was used instead of the “number of days per month when acute medications were used”. 

The 90 days with the highest number of prescribed acute medications were chosen for each patient and their prescription trends were analyzed. Prophylactic treatment was defined as more than one tablet prescription during the 90 days. Many prophylactic medications for headaches have various uses, including the treatment of hypertension, epilepsy, and depression. Therefore, it was impossible to determine whether these medications were prescribed specifically to prevent migraines or to treat other conditions.

### 2.2. Study 2 (longitudinal Study): Overprescription during the 2 Years from the Initial Diagnosis

We tracked the changes in prescription patterns over two years from the initial headache diagnosis to reveal changes in prescription patterns over time and to establish how many people were overprescribed to acute medication. We analyzed 845,470 patients aged 6–17 years from July 2010 to April 2022. The number of prescribed tablets was counted every 90 days from the initial headache diagnosis. Our analysis involved determining the percentage of patients who were overprescribed a combination of NSAIDs or triptans during the study period. We investigated various factors, including age, sex, and the point at which patients experienced overprescription of combination NSAIDs or triptans. We also calculated the amount of acute medication prescribed during the initial 90 days and the prophylactic medication prescribed within the same time frame.

We used a Cox regression analysis to identify the factors influencing the duration of overprescription of a combination of NSAIDs or triptans. No prescriptions for calcitonin-gene-related peptide (CGRP)-related drugs for migraine prevention were identified in the dataset during the specified periods.

### 2.3. Statistical Analysis

The normality of the data was assessed using the Shapiro–Wilk test. Variables that followed a normal distribution were presented as means (standard deviations), whereas those with a non-normal distribution were presented as medians (interquartile range). A log-minus-log plot was used to verify whether the assumption of proportional hazards was true. Statistical significance was established via a two-tailed *p* value < 0.05. The analysis used SPSS 29.0.0 (IBM Corp., Armonk, NY, USA) and Python 3.9.0, along with libraries including Pandas 2.0.2, PyCaret 3.1.0, scikit-survival 0.21.0, and Matplotlib 3.5.1.

### 2.4. Ethics

This study was approved by the Itoigawa General Hospital Ethics Committee (approval numbers: 2022-2, 2022-10). The need for written informed consent was waived due to the retrospective nature of the study. All procedures adhered to the principles outlined in the Declaration of Helsinki. Studies 1 and 2 followed the Strengthening the Reporting of Observational Studies in Epidemiology (STROBE) guidelines. 

## 3. Results

### 3.1. Study 1 (Cross-Sectional Study): Prescription Pattern for Headache Patients

Of 543,628 patients aged 6–17 years in the 2020 REZULT database, 62,568 (11.51%) were diagnosed with headaches via the health insurance system. Furthermore, 1524 of 62,568 (2.44%) patients aged 6–17 years received at least one prescription for acute medication in 2020.

Patients aged 6–11 years with headaches and a prescription for acute medication (*n* = 624/1524) had a mean age of 8.83 (±1.67) years, and 314/624 (50.32%) were female. A single NSAID (acetaminophen made up the majority) was the most frequently prescribed acute medication (620/624 patients, 99.36%), and triptans were prescribed to 5/624 (0.80%) patients. Most patients received only acute treatment (607/624 patients, 97.28%), and 17/624 (2.72%) patients received prophylactic treatment. The number of patients overprescribed with a combination of NSAIDs or triptans (≥30 tbl/90 d) was 0/624 (0%) and 1/624 (0.11%), respectively, (Table 1). 

Patients aged 11–17 years with headaches and a prescription for acute medication (*n* = 900/1524) had a mean age of 14.25 (±1.66) years, and 479/900 (53.22%) were female. A single NSAID (acetaminophen comprising the majority) was the most frequently prescribed acute medication (827/900 patients, 91.89%), and triptans were prescribed to 91/900 (10.11%) patients. Most patients received only acute treatment (824/900, 91.56%), whereas 76/900 (8.44%) received prophylactic treatment. The number of patients overprescribed with a combination of NSAIDs or triptans (≥30 tbl/90 d) was 6/900 (0.67%) and 10/900 (1.11%), respectively, (Table 2). 

Among the 6–17-year-old patients with headaches prescribed acute medication (*n* = 1524), triptans were prescribed to 96/1524 (6.30%) patients (Table 3). The mean number of triptan tablets over 90 days was 22.77 (±21.58). Prophylactic treatment was prescribed to 29/96 (30.21%). The number of patients who were overprescribed triptans (≥30 tbl/90 d) was 11/96 (11.46%), and the mean number of tablets was 38.82 (±14.19). Of these patients, 5/11 (45.45%) received prophylactic treatment.

### 3.2. Study 2 (Longitudinal Study): Overprescription during the Study Period

**Figure 1:**
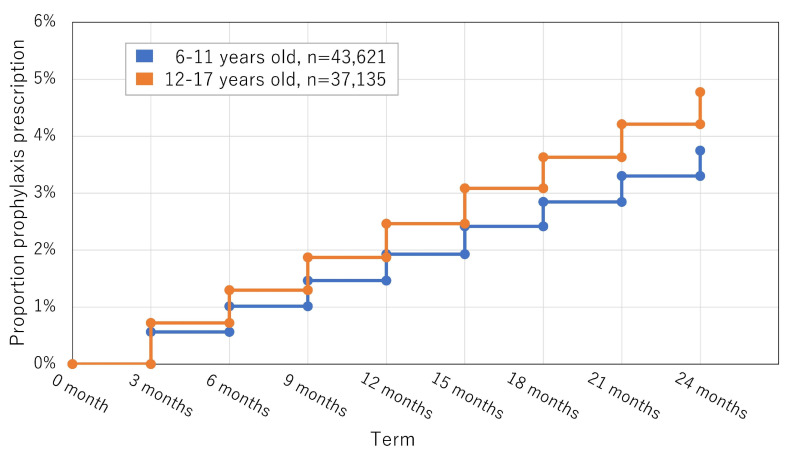
Kaplan–Meier curves for prescription of prophylactic treatment during the two years from the initial diagnosis. Trends in the prescription of prophylactic medication in patients with a persisting headache diagnosis for two years from the initial diagnosis.

Of the 845,470 patients aged 6–17 years from the REZULT database, 80,756/845,470 (9.55%) were first diagnosed with headaches that persisted for at least two consecutive years (July 2010 to April 2022). The mean age was 11.109 (±3.38) years, and 46,215 (57.23%) patients were women. During the first 90 days after headache diagnosis, 8/80,756 (0.01%) patients received >30 tablets of a combination of NSAIDs and 3/80,756 (0.004%) received >30 tablets of a triptan. Prophylactic medication was initiated in the first 90 days in 515/80,756 (0.64%) patients, which increased to 897/80,756 (1.11%) patients after two years. During the two years, 3408/80,756 (4.22%) patients with headaches were prescribed prophylactic medications on at least one occasion (Figure 1). The mean age was 11.51 (±3.15) years, and 1876/3408 (55.05%) were female. The mean duration of the first instance of prophylactic medication was 4.382 (2.32) terms of 90 days, approximately 12–15 months. Details of the sub-analyses of patients aged 6–11 years and 12–17 years are described in Table 4 and Table 5.

**Figure 2:**
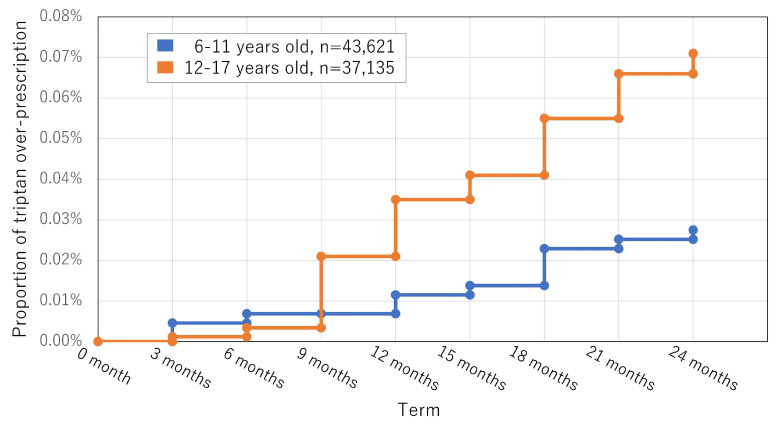
Kaplan–Meier curves for the overprescription of triptans during the two years from the initial diagnosis. Trends in overprescription in patients with a persisting headache diagnosis for two years from the initial diagnosis.

Of the 80 756 patients, 44 (0.05%) were overprescribed a triptan during the 2-year observation period. The mean age was 12.96 (±2.76) years, and 29/44 (65.91%) were female. The mean duration of the first instance of overprescription was 4.75 (1.93) terms of 90 days, approximately 12–15 months. Details of the sub-analyses of patients aged 6–11 years and 12–17 years are shown in Figure 2. 

Cox regression analysis revealed that more triptan prescriptions in the first 3 months were a risk factor for triptan overprescription in the 2 years among the 6–11-year-old patients (*p* < 0.001). The presence of single NSAID prescriptions and more triptan prescriptions during the first 90 days were risk factors for triptan overprescription in the 2 years among 12–17-year-olds (*p* < 0.001) (Table 6). 

## 4. Discussion

We used the REZULT database, a comprehensive health insurance database, to conduct two investigations into the prescription patterns of headache medications among individuals aged either 6–11 or 12–17 years. 

In the initial cross-sectional study, which involved 1524 patients aged 6–17 diagnosed with headaches and receiving prescribed medication in 2020, only 93 out of 1524 patients (6.102%) were prescribed prophylactic medication. Furthermore, 11 out of 1524 patients (0.72%) were identified as receiving an excessive prescription of triptan. For the subsequent longitudinal study, we examined 80,756 patients diagnosed with persistent headaches that lasted at least two consecutive years. During the two-year duration, 44 of 80,756 patients (0.05%) were found to have overprescribed triptans within 90 days at least once and 3408 of 80,756 patients (4.22%) received prescriptions for prophylactic medications at least once.

### 4.1. Investigation of Health Insurance Claim Databases of Adult Headache Disorders

Several reports have used health insurance claim databases to investigate prescription patterns for adult patients with migraines or headaches. Recently, triptan overprescription has been reported. An investigation of the Dutch Health Care Insurance Board Database of 2005, which included 46% of the total Dutch population, showed that triptan was used by 85,172/6.1 million patients (1.3%); of the prescriptions, 8844/85,172 (10.4%) were overprescribed. [23] A French study conducted in 2011 found that of 99,450 patients prescribed triptans, 2.26% (2243/99,450 patients) could potentially use them excessively. [28] Research conducted in 2012 in two regions of Italy showed that approximately 10% of individuals using triptan were categorized as frequent users, taking ten or more tablets per month. Among these frequent users, roughly two-thirds continued this pattern for another three months. Another study conducted in Austria, [25] which involved a large sample size of 5,918,487 individuals, revealed that 0.56% of the population were triptan users and 6.0% were identified as overusers. Triptan overusers tended to be older than those who did not overuse triptan. 

Similar to previous Japanese studies using health insurance claims data, Meyers et al. examined the treatment patterns of adult patients with migraines by analyzing information from the Japan Medical Data Center database. Over 3 years, among the 16,433 people diagnosed with migraine, 9873 (60.1%) were exclusively given acute medication, 3022 (18.4%) received prophylactic medications, and 3548 (21.6%) did not receive any prescribed medication. Additionally, a lower prevalence (1.4%) of migraine was observed than that observed in previous epidemiological studies. [1] This potential underestimate could arise from the fact that 69.4% of migraine patients had never sought medical advice for their headaches and only 11.6% of patients recognized their headaches as migraines. In this database, calcium-channel blockers and anticonvulsants have emerged as the predominant prophylactic medications.

Although triptan overuse has been studied using health claims data in adults, such studies have not yet been conducted in children and adolescents. In our study, the rate of triptan prescription (96/1524 headache patients with prescription (6.30%); 96/543,628 in the whole population (0.02%)) and the rate of overuse of triptans appeared to be lower than in adults. Our study suggests a modest potential for the use of triptans in children and adolescents compared to their efficacy in adults. In our study, prophylactic medication was prescribed to 93 patients (93/1524 headache patients with headache prescription (6.10%)). The proportion of prophylactic prescriptions was also lower than that in adults. The reason for this low prescription rate is unknown; it could be due to low efficacy, side effects that prevented prescription, or spontaneous headache remission.

Caution should be taken when interpreting prevalence and prescription rates. Patients with migraine resisted seeing a doctor [34], or if they did, they might not have been prescribed prophylactic medications [35]. Furthermore, Japanese herbal Kampo medicines [17] are the most commonly prescribed treatments. There are differences between the prophylactics that can be used in Japan and those available abroad [16].

### 4.2. Investigation of Health Insurance Claim Databases for Headache Disorders among Children and Adolescents

Law et al. [33] investigated the American Medical Expenditure Panel Survey data of 34,633 children aged 2–17 years, of whom 779 (2.6%) were headache-labeled between 2012 and 2015. The annual total healthcare expenditures were estimated to be 24.3% higher for headache patients than for other children and adolescents; therefore, they concluded that headaches in children and adolescents burden the economy.

Obermeier et al. [32] studied the medical costs of migraine treatment among children using the German health insurance claims data of 2597 (0.84%) children from a dataset of 306,926 children (6–11 years old) in 2017. They found that children with migraines had more comorbidities and that the annual medical costs for children with migraines were EUR 1018 compared with EUR 618 in the control group.

However, this study did not examine these economic health considerations [36,37]. In addition, the prevalence of migraine in health claims data may be lower than that reported in actual epidemiologic studies. In children, migraine patients may be either withheld from seeing a doctor or may not be diagnosed correctly, as these are also problems among adults.

### 4.3. Headache, Migraine, and MOH Prevalence in Children and Adolescents

Previous studies examining the prevalence of headaches, migraines, and MOH in children have been carried out. The prevalence rates vary notably among countries and ethnic groups. Nonetheless, our analysis of health insurance claims supports previous findings that both headaches and migraines become more frequent with age and are more commonly observed in females [38].

The global prevalence of MOH among children and adolescents appears to be <1%. The prevalence is 0.2% in Ethiopia [39], 0.7% in Lithuania and Mongolia [40,41], 0.8–1.2% in Zambia [42], 0.9% in Turkey [43], 1.1% in Iran [44], and 0.44% in Japan. [14] These investigations have revealed that MOHs are relatively infrequent in younger age groups but can occasionally manifest in adolescents. Children in need of medical care are likely taken to the hospital before they have access to their parents’ pain relievers, including OTC medications. Conversely, teenagers may not have constant parental oversight, which may lead to excessive OTC drug use because they purchase these medications independently.

Our study revealed that medical providers may cause MOH in patients by overprescribing a combination of NSAIDs or triptans. In particular, patients aged 15 years and older are seen in general practice rather than in pediatrics in Japan; therefore, medically induced MOH may be created in the same way as in adults [3]. Spreading correct knowledge about headache treatment [34] to pediatricians and physicians and using artificial intelligence support to improve the quality of care and diagnostic accuracy [45,46,47] may be solutions for reducing iatrogenic MOH. Furthermore, many existing prophylactic drugs are difficult to use in children due to their side effects. We hope that medications such as CGRP-related agents will become available for use in children in the future [48,49].

### 4.4. Limitations

This study had several limitations. First, a sampling bias occurred. For the cross-sectional study (Study 1), we specifically chose a 90-day timeframe with the highest number of acute medication prescriptions (tablet counts). In the longitudinal study (Study 2), we focused on patients who were diagnosed with headaches for two years following their initial diagnosis. This approach may have led to the inclusion of a significant proportion of patients with severe headaches. Additionally, as NSAIDs are not exclusively prescribed for headaches, excluding patients who regularly receive analgesics for other forms of persistent pain, such as back pain, was impossible. Our selection of patients who consistently bore the label “headache”, aimed to identify and encompass as many instances of recurrent headaches as possible. Nonetheless, it is essential to note that secondary headaches, which can arise from factors such as common cold, physical trauma [50], and arterial dissection [51,52], were not eliminated. Consequently, any interpretations drawn from these findings should be interpreted with caution. 

Second, we retrospectively analyzed billing-related administrative claims data. Patient identification relied solely on available database information, preventing us from assessing migraine severity, precise daily medication intake, and utilization of OTC drugs. The calculation of a single NSAID’s exact dosage and the frequency of individual NSAIDs was impossible. Certain migraine therapies have diverse applications (e.g., anticonvulsants and antidepressants), leading to potential misclassification where patients might have been inaccurately categorized as receiving migraine treatment even if the treatment was intended to address another coexisting condition. Third, we could not track patients using insurance systems that were not included in the REZULT database. 

Fourth, the exact isolation of patients exclusively experiencing migraines proved unfeasible due to the grouping of various headache types within a single category. Subsequent reevaluation focusing solely on migraines is a potential avenue for exploration. The observable frequency of headache-related conditions might have increased because of diagnostic assignments made for insurance purposes, with instances where ailment labels were retained or inadvertently omitted from removal. Correspondingly, the proportion of prescribed medications could have been inaccurately underestimated in cases where medical practitioners failed to designate migraines when explicitly prescribing triptan. Consequently, prudent consideration is needed when interpreting prevalence statistics.

Fifth, we could not evaluate patients with migraines who were not prescribed any medication. In addition, prescription patterns vary from country to country and topiramate and other alternative treatments [53] were not tracked in the current data. In addition, Japanese herbal Kampo medicines cannot be prescribed [16,28,54,55,56,57,58,59,60]. Finally, we will continue to monitor the alterations in this prescription trend in response to heightened headache awareness [34] and the advent of novel medications, including CGRP-related drugs [49,61,62] and lasmiditan [63,64].

## 5. Conclusions

We analyzed the prescription patterns for headaches using the REZULT database, a large health insurance database. Our findings indicated that of 1524 patients diagnosed with headaches, only 6.10% (93/1524) were prescribed prophylactic medications. Among the 1524 patients who received acute medications (NSAIDs or triptans), 11/1524 (0.72%) were identified as having triptans overprescribed. In our two-year longitudinal study involving 80,756 patients, 44/80,756 (0.05%) were found to have received excessive triptan prescriptions at least once during a 90-day period and 3408/80,756 (4.22%) patients with headache were prescribed prophylactic medications within a 90-day timeframe on at least one occasion. This study is the first to examine treatment patterns for headaches in children in detail from insurance claim data.

## Figures and Tables

**Table 1:** Treatment pattern for headache patients aged 6–11 years.

Characteristics	Overall	%/SD	A w/o P	%/SD	T w/o P	%/SD	A w/P	%/SD	T w/P	%/SD
**Patients number**	624	100%	607	97.28%	4	0.64%	17	2.72%	1	0.16%
**Number of triptan overprescriptions (%)**	1	0.11%	1	0.17%	1	25.00%	0	0%	0	0%
**Age (mean, SD)**	8.83	1.67	8.81	1.67	10.25	0.25	9.47	1.67	10	-
**Sex: Female**	314	50.32%	305	50.25%	3	25.00%	9	52.94%	1	100%
**Acute treatment**										
Combination NSAIDs	1	0.16%	1	0.17%	-	-	0	0%	-	-
Single NSAIDs	618	99.04%	602	99.18%	-	-	16	94.12%	-	-
Triptans	3	0.48%	2	0.33%	2	50.00%	1	5.88%	1	100%
Single NSAIDs and triptans	2	0.32%	2	0.33%	2	50.00%	0	0%	-	-
**Prophylactic treatment**	17	2.72%	-	-	-	-	17	100%	1	100%
Calcium-channel blockers (lomerizine)	1	0.16%	-	-	-	-	1	5.88%	-	-
Antidepressants (amitriptyline)	1	0.16%	-	-	-	-	1	5.88%	-	-
Japanese herbal Kampo medicine	14	2.24%	-	-	-	-	14	82.35%	1	100%
Anticonvulsants and Kampo medicine	1	0.16%	-	-	-	-	1	5.88%	-	-

A, acute treatment; NSAIDs, nonsteroidal anti-inflammatory drugs; P, prophylactic treatment; SD, standard deviation; T, triptans.

**Table 2:** Treatment pattern for headache patients aged 12–17 years.

Characteristics	Overall	%/SD	A w/o P	%/SD	T w/o P	%/SD	A w/P	%/SD	T w/P	%/SD
**Patients number**	900	100%	824	91.56%	63	7.00%	76	8.44%	28	3.11%
**Number of combination NSAID overprescriptions (%)**	6	0.67%	5	0.61%	0	0.00%	0	0.00%	1	3.57%
**Number of triptan overprescriptions (%)**	10	1.11%	5	0.61%	5	7.94%	5	6.58%	5	17.86%
**Age (mean, SD)**	14.25	1.66	14.20	1.65	14.16	1.63	14.76	1.65	15.11	1.63
**Sex: Female**	479	53.22%	428	51.94%	39	61.91%	51	67.11%	20	71.43%
**Acute treatment**										
Combination NSAIDs	13	1.44%	10	1.21%	-	-	3	3.95%	-	-
Single NSAIDs	793	88.11%	749	90.90%	-	-	44	57.90%	-	-
Triptans	55	6.11%	42	5.10%	42	66.67%	13	17.11%	13	46.43%
Combination NSAIDs and single NSAIDs	3	0.33%	2	0.24%	-	-	1	1.32%	-	-
Single NSAIDs and triptans	31	3.44%	18	2.18%	18	28.57%	13	17.11%	13	46.43%
All 3 types	5	0.56%	3	0.36%	3	4.76%	2	2.63%	2	7.14%
**Prophylactic treatment**	76	8.44%	-	-	-	-	76	100%		100%
Calcium-channel blockers (lomerizine)	17	1.89%	-	-	-	-	17	22.37%	10	35.71%
Beta-blockers (propranolol)	1	0.11%	-	-	-	-	1	1.32%	1	3.57%
Anticonvulsants (valproic acid)	15	1.67%	-	-	-	-	15	19.74%	5	17.67%
Antidepressants (amitriptyline)	3	0.33%	-	-	-	-	3	3.95%	3	10.71%
Japanese herbal Kampo medicine	27	3.00%	-	-	-	-	27	35.53%	1	3.57%
Combination of 2 types	12	1.33%	-	-	-	-	12	15.79%		28.57%
Combination of 4 types	1	0.11%	-	-	-	-	1	1.32%		0%

A, acute treatment; d, days; NSAIDs, nonsteroidal anti-inflammatory drugs; P, prophylactic treatment; SD, standard deviation; T, triptans; tbl, tablets.

**Table 3:** Treatment pattern of triptans for 6–17-year-old patients.

Characteristics	Overall	%/SD	0–11 tbl/90 d	%/SD	12–29 tbl/90 d	%/SD	30–44 tbl/90 d	%/SD	45– tbl/90 d	%/SD
**n (all = 1524)**	96	100%	64	66.67%	21	21.88%	9	9.38%	2	2.08%
**Number of tbl/90 d**	22.77	21.58	6.78	2.85	19571	5.56	35.75	5.06	62.50	-
**Age (mean, SD)**	14.23	1.80	14.14	1.72	14.43	1.71	14.44	1.77	14.00	-
**Sex: Female**	60	62.50%	38	59.38%	11	52.38%	9	100%	2	100%
**Acute treatment**										
Triptans	58	60.42%	40	62.50%	14	66.67%	3	33.33%	1	50%
Single NSAIDs and triptans	33	34.38%	21	32.81%	5	23.81%	6	66.67%	1	50%
All 3 types	5	5.21%	3	4.69%	2	9.52%	0	0%	0	0%
**Prophylactic treatment**	29	30.21%	9	14.06%	15	71.43%	4	44.44%	1	50%
Calcium-channel blockers (lomerizine)	10	10.42%	4	6.25%	4	19.05%	1	11.11%	1	50%
Beta-blockers (propranolol)	1	1.04%	1	1.56%	0	0%	0	0%	0	0%
Anticonvulsants (valproic acid)	5	5.21%	1	1.56%	4	19.05%	0	0%	0	0%
Antidepressants (amitriptyline)	3	3.13%	1	1.56%	0	0%	2	22.22%	0	0%
Japanese herbal Kampo medicine	2	2.08%	2	3.13%	0	0%	0	0%	0	0%
Combination of 2 types	8	8.33%	0	0%	7	33.33%	1	11.11%	0	0%

d, days; NSAIDs, nonsteroidal anti-inflammatory drugs; SD, standard deviation; tbl, tablets.

**Table 4:** Changes in prescribing patterns over time for patients aged 6–11 years (*n* = 43,621).

Variables	1–3 m	4–6 m	7–9 m	10–12 m	13–15 m	16–18 m	19–21 m	22–24 m
**No prescription**	86.70%	89.95%	88.70%	87.98%	88.05%	88.18%	86.63%	86.58%
**Acute treatment**	12.92%	9.68%	10.90%	11.55%	11.43%	11.28%	12.78%	12.79%
Single NSAIDs	12.88%	9.65%	10.84%	11.50%	11.37%	11.19%	12.68%	12.69%
Combination NSAIDs	0.02%	0.01%	0.02%	0.01%	0.01%	0.03%	0.03%	0.03%
Triptans	0.05%	0.05%	0.06%	0.05%	0.08%	0.11%	0.13%	0.14%
Combination NSAIDs ≥ 30 tbl/90 d	0.01%	0%	0%	0%	0%	0%	0.01%	0%
Triptans ≥ 30 tbl/90 d	0.01%	0%	0%	0.01%	0%	0.01%	0.01%	0%
**Both acute and prophylactic treatments**	0.16%	0.17%	0.20%	0.18%	0.26%	0.23%	0.24%	0.31%
**Prophylactic treatment**	0.54%	0.53%	0.61%	0.64%	0.78%	0.77%	0.83%	0.94%
Calcium-channel blockers (lomerizine)	0.01%	0.01%	0.01%	0.02%	0.03%	0.03%	0.04%	0.06%
Beta-blockers (propranolol)	0.01%	0.01%	0.00%	0.01%	0.01%	0.02%	0.01%	0.02%
Anticonvulsants (valproic acid)	0.03%	0.07%	0.10%	0.11%	0.15%	0.19%	0.23%	0.26%
Antidepressants (amitriptyline)	0.02%	0.02%	0.02%	0.02%	0.03%	0.03%	0.03%	0.04%
Japanese herbal Kampo medicine	0.48%	0.43%	0.49%	0.49%	0.58%	0.51%	0.54%	0.59%

d, days; m, months; NSAIDs, nonsteroidal anti-inflammatory drugs; tbl, tablets.

**Table 5:** Changes in prescribing patterns over time for patients aged 12–17 years (*n* = 37,135).

Variables	1–3 m	4–6 m	7–9 m	10–12 m	13–15 m	16–18 m	19–21 m	22–24 m
**No prescription**	87.91%	92.17%	91.25%	90.87%	90.91%	90.89%	90.56%	90.47%
**Acute treatment**	11.66%	7.38%	8.20%	8.54%	8.39%	8.46%	8.69%	8.74%
Single NSAIDs	11.32%	7.13%	7.90%	8.21%	8.03%	8.04%	8.26%	8.35%
Combination NSAIDs	0.14%	0.09%	0.08%	0.09%	0.11%	0.11%	0.14%	0.12%
Triptans	0.33%	0.28%	0.38%	0.36%	0.41%	0.44%	0.48%	0.47%
Combination NSAIDs ≥ 30 tbl/90 d	0.02%	0.01%	0.01%	0.01%	0.02%	0.03%	0.02%	0.02%
Triptans ≥ 30 tbl/90 d	0.00%	0.00%	0.02%	0.02%	0.02%	0.02%	0.02%	0.01%
**Both acute and prophylactic treatments**	0.32%	0.27%	0.32%	0.41%	0.37%	0.47%	0.47%	0.52%
**Prophylactic treatment**	0.75%	0.71%	0.88%	1.00%	1.07%	1.12%	1.23%	1.31%
Calcium-channel blockers (lomerizine)	0.06%	0.08%	0.14%	0.16%	0.15%	0.17%	0.18%	0.24%
Beta-blockers (propranolol)	0.01%	0.02%	0.03%	0.04%	0.05%	0.05%	0.04%	0.05%
Anticonvulsants (valproic acid)	0.08%	0.13%	0.19%	0.24%	0.26%	0.32%	0.37%	0.39%
Antidepressants (amitriptyline)	0.02%	0.02%	0.04%	0.07%	0.09%	0.09%	0.08%	0.08%
Calcium-channel blockers (verapamil)	0%	0%	0%	0.01%	0.01%	0.01%	0.01%	0.01%
Japanese herbal Kampo medicine	0.60%	0.50%	0.52%	0.54%	0.58%	0.57%	0.62%	0.64%

d, days; m, months; NSAIDs, nonsteroidal anti-inflammatory drugs; tbl, tablets.

**Table 6:** Cox regression analyses results for triptan overprescription.

6–11-Year-Olds, *n* = 43,621, Event = 12 (0.0275%)	B	SE	Wald	*p*-Value	Odds	95%CI (Lower)	(Upper)
Female	0.04	0.68	0.00	0.960	1.04	0.26	3.90
Age (y.o.)	0.40	0.22	3.15	0.076	1.49	0.96	2.30
Presence of a prescription of single NSAID during the first 90 days	−0.27	1.06	0.06	0.803	0.77	0.10	6.15
Combination NSAIDs during the first 90 days (tbl/90 d)	−5.86	14,794.23	0.00	0.999	0.00	0.00	Inf.
Triptans during the first 90 days (tbl/90 d)	0.46	0.06	63.94	<0.001	1.58	1.41	1.77
Presence of a prophylactic treatment prescription during the first 90 days	−12.83	674.21	0.00	0.985	0.00	0.00	Inf.
12–17-Year-Olds, *n* = 37,135, Event = 32 (0.0862%)	B	SE	Wald	*p*-Value	Odds	95%CI (Lower)	(Upper)
Female	0.60	0.45	1.81	0.178	1.82	0.76	4.38
Age (y.o.)	−1.47	51.40	0.00	0.98	0.23	0.00	1.30 × 10^43^
Presence of a prescription of a single NSAID during the first 90 days	0.16	0.03	40.79	<0.001	1.17	1.12	1.23
Combination NSAIDs during the first 90 days (tbl/90 d)	2.39	0.58	17.07	<0.001	10.87	3.50	33.70
Triptans during the first 90 days (tbl/90 d)	0.48	0.40	1.47	0.225	1.62	0.74	3.51
Presence of a prophylactic treatment prescription during the first 90 days	−0.02	0.11	0.05	0.824	0.98	0.79	1.21

B, slope; CI, confidence interval; d, days; SD, standard deviation; SE, standard error; tbl, tablets; NSAIDs, nonsteroidal anti-inflammatory drugs.

## Data Availability

The datasets used and/or analyzed during the current study are available from the corresponding author on reasonable request.

## Supplementary Materials

The following supporting information can be downloaded at: https://www.mdpi.com/article/10.3390/life14010096/s1, Supplementary File S1: the codes for the drug price list (Excel file).

## Abbreviations

CGRP	calcitonin-gene-related peptide
CI	confidence interval
ICD-10	International Statistical Classification of Diseases and Related Health Problems-10
ICHD-3	International Classification of Headache Disorders, 3rd edition
NSAIDs	nonsteroidal anti-inflammatory drugs
OTC	over-the-counter
TTH	tension-type headache
